# A brief historic overview of sexual and gender diversity in neuroscience: past, present, and future

**DOI:** 10.3389/fnhum.2024.1414396

**Published:** 2024-09-13

**Authors:** Jhon Alexander Moreno, Riccardo Manca, Lucas Albrechet-Souza, Juan A. Nel, Ioannis Spantidakis, Zindi Venter, Robert-Paul Juster

**Affiliations:** ^1^Department of Psychology, Université de Montréal, Montreal, QC, Canada; ^2^Centre de recherche de l'institut universitaire de gériatrie de Montréal, CIUSSS du Centre-Sud-de-l'Île-de-Montréal, Montreal, QC, Canada; ^3^Notre-Dame Hospital, Centre intégré universitaire de santé et de services sociaux du Centre-Sud-de-l'Île-de-Montréal (CCSMTL), Montreal, QC, Canada; ^4^Department of Life Sciences, Brunel University London, Uxbridge, United Kingdom; ^5^Department of Medicine and Surgery, University of Parma, Parma, Italy; ^6^Department of Cell Biology and Anatomy, Louisiana State University Health Sciences Center, New Orleans, LA, United States; ^7^Department of Psychology, University of South Africa, Pretoria, South Africa; ^8^Department of Psychology, Scientific College of Greece, Athens, Greece; ^9^Department of Psychiatry and Addiction, Université de Montréal, Montreal, QC, Canada

**Keywords:** key developments, LGBTQIA+, neuroscience, literature review, research trends, sexual and gender diversity, SOGI, LGBTQIA+ history

## Introduction

For decades, scholars across disciplines, including researchers, scientists, and clinicians, have exhibited a keen interest in exploring the neuroscientific underpinnings of sexual and gender diversity. The purpose of this paper is thus to provide an overview and opinion on the evolution of related research. To achieve this, the authors conducted a bibliographic search of the literature in MEDLINE and PsycInfo from inception to September 2023 with specific keywords, including “neuroscience,” and variations of “lesbian, gay, bisexual, transgender, queer, intersex, asexual, and people with other sexual orientations and forms of gender expression (LGBTQIA+).”

**Figure 1:**
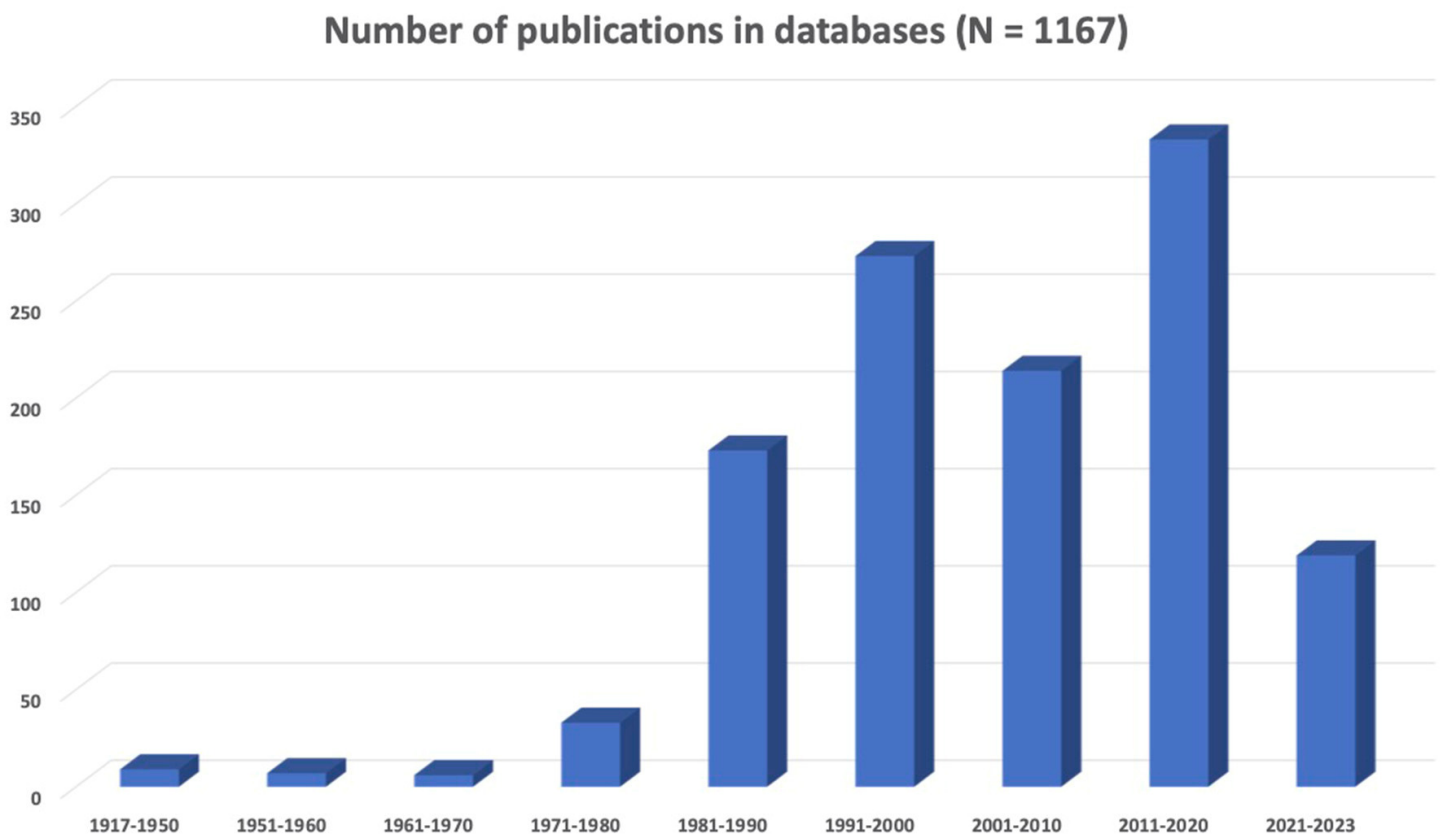
Summary of the scientific literature about neuroscience and LGBTQIA+ issues.

The results presented in Figure 1 show that there is an increase in neuroscience and LGBTQIA+ concerns represented in the scientific literature. In particular, the decade from 1991 to 2000 saw a significant growth in related publications, with the field of neuroscience establishing itself as distinct from psychiatry. However, 2011–2020 was the most prolific decade in the history of sexual and gender diversity in neuroscience. In summary, there were 1,167 records (i.e., scientific articles, book chapters, opinion letters, among others) corresponding to a collective sample of 444,249 individuals on the LGBTQIA+ spectrum. Most research centered on gay men (32%) or transgender people (24.3%), and only a few addressed lesbian (1.1%), bisexual (1.3%), or other identities (1.1%). When mixed samples were included, the majority corresponded to gay and lesbian people (15.3%). The most common topics were brain development (34.6%), general biological aspects (31.8%), and human immunodeficiency virus (HIV)-related research (27.5%). In addition, articles focused on social (19%), psychological (15.9%), and neuropsychological (13.3%) aspects of sexual and gender identity. Animal studies accounted for 7.8% of research.

Before considering the future of neuroscience of sexual and gender diversity, we first provide a chronological overview of select related key developments in the field.

## Overview of select key developments in sexual and gender diversity in neuroscience

### Early work

The first record we identified appeared in 1917 in the context of mental disorders suggesting that brain pathology could be related with sexual behavior (Meyer, 1917). Until 1950, only nine publications captured by this search focused on human sexuality, and as complex and multi-determined by biological, psychological, and sociological aspects (Morselli, 1931). This literature indicated that structural damages have an impact on sexual behavior. For instance, “bad thyroid gland may cause queer behavior in the human” (Moss, 1932). During the same period, homosexuality was, in fact, classified as part of psychiatric syndromes (Rosanoff, 1938) and this continued to be the case until 1973. Lobotomy was offered as an option for treating the so-called “sex psychopaths” (Banay and Davidoff, 1942).

From 1951 to 1960, seven publications addressed topics linking sexuality to brain abnormalities. For instance, records theorized upon associations linking disturbed cerebral functioning with bisexuality and schizophrenia (Potzl, 1951) and the Klüver-Bucy syndrome with “homosexual advances” following the removal of the temporal lobes (Terzian and Dalle Ore, 1955). Later, scientific research on the same syndrome indicates that it is characterized by increased nonselective sexual behavior, and not homosexuality itself. However, it was interpreted as homosexual behavior at the time the study was conducted. Between 1961 and 1970, six records were found on topics such as gender identity in gay males and psychosexual functioning of the brain (Money, 1965), the use of hypothalamotomy for sexual disorders (Anonymous, 1969), and literature suggesting a link between criminality and psychiatric disorders, with homosexuality being considered one of them (Guze et al., 1969).

### Redefining research and classification systems (1970s and 1990s)

Informed by changing social and scientific attitudes and theories about sexual orientation, in 1973, homosexuality as a mental disorder was officially removed from the Diagnostic and Statistical Manual of Mental Disorders (DSM). From 1971 to 1980, 33 records included topics studying the use of electroencephalographic techniques to document pleasure in men (Heath, 1972) and transgender people (Nussselt and Kockott, 1976), as well as brain functioning differences between gay men and heterosexual men (Wilson et al., 1973).

During this time, neurochemistry studies flourished. This included the role of brain monoamines in male sexual behavior (Gessa and Tagliamonte, 1974), the role of hormones as an explanation for homosexuality in men (Dorner et al., 1975), and animal models of homosexuality in rats and rabbits that experimented on serotonin levels (Fratta et al., 1977). Brain injury studies included the link between homosexuality and early brain damage (Holzer, 1976) and there were studies linking “latent homosexuality” with schizophrenia in males (Sigal, 1978). In addition, we find the first reference to “patients with the problem of intersexuality” when endocrine problems can “masculinize” females and “demasculinize” males (Ehrhardt and Meyer-Bahlburg, 1979).

From the period between 1981 and 1990, the search led to 173 records. And yet, there was only one study addressing neuroendocrine contributions to male and female homosexuality (MacCulloch and Waddington, 1981). A few studies also provided a more nuanced approach and considered sex-related brain differences as a mosaic of both male and female “characteristics” influenced by hormones (Neumann and Elger, 1981). The 1980s also marked the beginning of investigations on samples of gay men to document opportunistic infections of the central nervous system associated with HIV (Anderson et al., 1983; Handler et al., 1983).

Despite changes in psychiatric nomenclature, psychopathological views persisted. For instance, a study suggested a relationship between decreased serotonergic activity and “delusional ideas of homosexual content” in individuals with paranoid schizophrenia (Rinieris et al., 1985). Yet, during this decade, researchers also started to question studies focusing on the biological basis of homosexuality as tainted by personal beliefs and cultural prejudices (Ricketts, 1984). Furthermore, the traditional nature/nurture debate around the genesis of sexual behaviors (homosexual, bisexual, and heterosexual) was enriched by new concepts, such as “critical-period/nurture” in brain development and social influences on behavior (Money, 1986). Another study suggested a change in sexual preference following brain injuries involving limbic system structures (Miller et al., 1986).

More integrative theories started to appear classifying sexual orientation as a complex phenomenon determined by genetic-hormonal, pharmacological, maternal stress, immunological, and social experiential variables (Ellis and Ames, 1987). An integrative book consolidating multiple perspectives to explain sexual orientation was published during this decade (Money, 1988). Interestingly, this period was also the beginning of theories linking a cluster of cells located in the preoptic area of the hypothalamus with sexual orientation and gender identity (Swaab and Hofman, 1988).

The decade from 1991 to 2000 showed a significant explosion in scientific productivity as compared to the previous decades with 273 records. A new clinical interest started to emerge leading to neurorehabilitation approaches specifically tailored for the needs of gay and lesbian people with brain injuries (Mapou, 1990). Investigating the neural correlates of sexual orientation and gender identity were not as important anymore as finding alternatives to meet LGBTQIA+ health and wellness needs following a brain injury.

With the proliferation of neuroscience emerging as a field separate from psychiatry, the results of neuroanatomical and neuropsychological studies began to be used in perspectives that were less pathologizing. In this manner, there were studies suggesting a neurobiological component related to hemispheric functional lateralization with an over-representation of left-handedness in gay men and lesbian women (McCormick et al., 1990). The results of a neuroanatomical study also showed that the midsagittal plane of the anterior commissure in gay men was 18% larger than in heterosexual women and 34% larger than in heterosexual men (Allen and Gorski, 1992). In addition, the number of cells in the suprachiasmatic nucleus of the hypothalamus in gay men was indicated as twice as large in comparison to heterosexual individuals (Swaab et al., 1995), while a female-sized volume of the central subdivision of the bed nucleus of the stria terminalis was found in male-to-female transsexuals^1^ (Zhou et al., 1997; Kruijver et al., 2000).

Critiques of genetic (Hamer and Copeland, 1994) and hormonal studies (Garnets and Kimmel, 1993) concerning human sexual orientation started to appear during this time. This perspective essentially argued against “nature” as a single biological determinant of sexual orientation (Byne and Parsons, 1993; Banks and Gartrell, 1995; Gooren, 1995) and instead considered a multitude of biopsychosocial factors (Friedman and Downey, 1993; Bancroft, 1994; Doell, 1995; Looy, 1995; Byne, 1997). These changes were also reflected in animal models of homosexuality with an alternative bio-social program of research on the development of sexual behavior in animals (Fausto-Sterling, 1995). Notwithstanding, genetic studies with Drosophila models of sexual orientation continued to flourish (Ferveur et al., 1995; Ito et al., 1996; Yamamoto et al., 1996). There were also animal models of homosexuality in rams and sheep (Perkins and Fitzgerald, 1997), and rat models of bisexuality (Aron, 1999).

Critics also called attention to the fact that this research excluded females/women and ethnic minorities and denied the political, cultural, and historical dimensions of sexuality (Hegarty, 1997). Reflecting a refocus on care, neuropsychological literature in humans insisted on the exploration with the client and their support system and how their sexual orientation affected assessment and rehabilitation (Morales, 2000). Also, when a neuropsychological assessment was conducted, the sexual orientation of the individual influenced outcomes and treatment when external psychosocial stressors were considered in rehabilitation. Those stressors became commonly known as “minority stress” caused by stigma, prejudice, and discrimination being responsible for a sustained unwelcoming stressful social environment having a deleterious impact on the health of sexually and gender-diverse people.

### Increasing criticism to binarism in neuroscientific research

Between the decade of 2001 and 2010, the search identified a total of 214 records. Toward the end of this decade, there was a scientific debate about the removal of gender identity disorders as formal diagnoses in both the DSM and the World Health Organization (WHO) International Classification of Diseases (ICD) (Bockting, 2009). The following examples show that the interest during this time was in demonstrating the anatomical and hormonal differences in sexually and gender-diverse individuals. It is also important to note concerns regarding issues of replication. Indeed, studies regarding differences in the anterior commissure of the brain could not be replicated (Lasco, 2001). A study concluded that compared with heterosexual women, lesbians display less gray matter bilaterally in the temporo-basal cortex, ventral cerebellum, and left ventral premotor cortex (Ponseti et al., 2007).

A few reports were also published on the common occurrence of phantom genitalia following gender confirmation surgery, with a post-surgical incidence of 30% of phantom penises following gender confirmation surgery in female-to-male transgender participants, as compared to 60% in men following penectomy for cancer treatment (Ramachandran and McGeoch, 2007, 2008). A study found the sex reversal of one of the interstitial nuclei of the anterior hypothalamus in transgender people (Garcia-Falgueras and Swaab, 2008). Studies investigating the activation of the hypothalamus when participants were watching erotic videos found a lack of hypothalamic activation and intense autonomic response following exposure to videos of the “opposite” sexual orientation (Paul et al., 2008). A positron emission tomography (PET)-magnetic resonance imaging (MRI) study indicated sex-atypical cerebral asymmetry and functional connections in homosexual subjects showing sex-atypical amygdala connections (Savic and Lindstrom, 2008).

Other research during this time suggested that the isthmal area corresponding to the posterior region of the callosal body connecting parieto-temporal cortical regions was larger in gay men and even predicted 96% of sexual orientation in men based on neuroanatomical and cognitive variables (Witelson et al., 2008). Also, a study showed that male-to-female (MTF) transsexuals showed a significantly larger volume of regional gray matter in the right putamen compared to cisgender men (Luders et al., 2009).

There was increasing debate on the way science had contributed to the discussion on sex differences. Indeed, distorted or exaggerated evidence sometimes reflected social and political opinions (Rogers, 2001), interests, and values (Saravi, 2007). During this time, neuroscientists discussed the social, forensic, and therapeutic implications of their findings (Wolpe, 2004). Neuropharmacological approaches included a study on the differential cerebral response of antidepressants in gay and heterosexual men (Kinnunen, 2002), and the effect of prenatal exposure to therapeutic drugs on brain feminization/demasculinization (Ellis and Hellberg, 2005).

A neuroimmunological theory emerged as the “maternal immune hypothesis.” Similar to the fraternal birth order effect, this model suggested that homosexuality in human males was predicted by higher numbers of older brothers reflecting the progressive immunization of some mothers to male-specific antigens and their effects on the sexual differentiation of the brain (Blanchard, 2004; Blanchard and Bogaert, 2004). In the meantime, intersex individuals were studied under the perspective of hormonal abnormalities (Hines, 2004a,b). Notably, female-to-male transsexuals (FTM) tested before and after 6 months of androgen treatment significantly improved their performance on a visual memory task (Gomez-Gil et al., 2009), with an fMRI study showing that differences in activation patterns remained stable over the course of hormonal treatment (Schoning et al., 2010).

### A new era of reformulation in research and classification systems

The decade from 2011 to 2020 included 333 records, which is the most prolific decade in the history of sexual and gender diversity in neuroscience to date. Transgender-related diagnoses were removed from the ICD (11^th^ edition) chapter on mental and behavioral disorders in 2018, taking effect clinically in 2022. Longitudinal case reports of positive effects of hormonal treatment in adolescents (Cohen-Kettenis et al., 2011) and the conviction that a person's sexual orientation arises in large part from biological processes that are already underway before birth led scientists to increasingly see sexual and gender diversity as something to be valued, celebrated, and welcomed into society (LeVay, 2011).

An increasing recognition emerged that monocausal explanations were unable to effectively address the complexity of transgender identity development and the integration of neurobiological findings into other disciplines were deemed to be the best avenue forward (Nieder et al., 2011). As such, it was recommended that genetic, neuroendocrinological, neurostructural, and neurofunctional findings must be integrated within a multidisciplinary framework to reach a more comprehensive vision of transgenderism. Still, diffusion tensor imaging showed that the white matter microstructure pattern in untreated MTF transgender participants fell halfway between the pattern of male and female cisgender individuals (Rametti et al., 2011). A fMRI study in FTM transsexuals showed that making a brain “more male” by the application of androgens not only reduced the activity of a core neural hub (frontal, temporal, and striatal regions), but also altered the organization of the brain network with increased connectivity among limbic regions supporting emotional and social cognitive processes related to empathy and mentalizing (Ye et al., 2011). MTF transsexuals showed significantly larger gray matter volume in the right putamen compared to cisgender men (Luders et al., 2012). Another study ascertained that FTM participants showed evidence of subcortical gray matter “masculinization,” while MTF individuals showed evidence of cortical thickness “feminization” (Zubiaurre-Elorza et al., 2013), but these findings were not replicated in non-Western samples (Sorouri Khorashad et al., 2020). Compared to heterosexual men, gay men and heterosexual women had similar thickness values in visual cortices and thalamic volumes (Abe et al., 2014). A study showed decreased hemispheric connectivity ratios of subcortical/limbic areas for both MTF and FTM transgender groups (Hahn et al., 2015).

The activation of regions within the temporo-parietal junction linked to empathy was also observed in individuals sexually attracted to men (heterosexual women and gay men) showing greater empathy levels than participants attracted to women (heterosexual men and lesbians) (Perry et al., 2013). Other identities such as bigender appeared in the context of medical literature referring to brain plasticity (Case and Ramachandran, 2012). A call for a feminist/queer critical neuroscience framework based on interdisciplinarity emerged to address controversies in the literature (Kraus, 2012). The first reports of a link between individuals living on the autism spectrum with gender diversity (Jones et al., 2012; Pasterski et al., 2014) as well as the neurodiversity movement (Jaarsma and Welin, 2012; Bertilsdotter Rosqvist et al., 2020) appeared in this decade. Good summaries addressing both ultimate (e.g., evolutionary) and proximate causes influenced the spectrum of sexual orientations (Hill et al., 2013). In terms of cognitive abilities and brain activation patterns, a study showed higher verbal fluency scores in FTM adolescents as compared to MTF adolescents and cisgender boys and girls, with no significant brain activation differences (Soleman et al., 2013). Genetic research suggested the influence of the sex hormone-related genes, estrogen receptor beta (ERbeta) in the “defeminization” of the female brain in FTM individuals (Fernández et al., 2014).

A focus shift on social determinants of health brought research documenting the deleterious effect of structural stigma causing a chronic activation of the hypothalamic-pituitary-adrenocortical axis in LGB young adults who were raised in highly stigmatizing environments as adolescents evidencing a blunted cortisol response (Hatzenbuehler and McLaughlin, 2014). In addition, more sophisticated techniques continued to address functional and neuroanatomical differences by gender diversity. For instance, a study showed that a rightward asymmetry of the serotonin transporter distribution observed via PET imaging in the midcingulate cortex of cisgender males was absent in females and MTF transsexuals (Kranz et al., 2014). Electroencephalogram (EEG) and event-related potentials to study implicit levels of discomfort toward homosexuality showed differences in the processing of visual images that occur as early as 200 milliseconds and may be moderated by familiarity in heterosexual participants (Dickter et al., 2015). A study on white matter microstructure suggested that the neuroanatomical signature of transgenderism was related to brain areas processing self-perception and body ownership, whereas homosexuality seemed to be associated with less pronounced cerebral sexual differentiation of white matter tracts (Burke et al., 2017; Manzouri et al., 2017). A study showed different functional connectivity patterns in the brains of transgender compared with cisgender girls, boys, and adolescents (Nota et al., 2017). New developments included a model to explain functional neuroimaging correlates of transgender identity development and gender dysphoria (Altinay and Anand, 2020). Animal models of homosexuality became available with the use of aromatase inhibitors affecting the estrogen synthesis during the critical periods of brain sexual differentiation (Olvera-Hernandez and Fernandez-Guasti, 2015).

During this decade, critics focused on disparities involving LGBT communities who remained understudied in medicine, including neurology (Rosendale and Josephson, 2015). The rationale behind this argument seemed based on a binary paradigm of sexual and gender diversity disregarding the effects of minority stress and creating limitations to culturally competent care in medicine. Even when minority stress was taking a more important place in the neuroscience of sexual and gender diversity, there were reports suggesting a direct link with biological aspects as a case report of a transgender person that became cisgender following a status epilepticus (Parkinson, 2015).

In another study, heterosexual women and gay men showed more left-brain lateralization for processing female faces as compared to heterosexual men with more right-brain lateralization (Rahman and Yusuf, 2015). Neuropsychological studies indicated that compared to heterosexual men, heterosexual women and gay men showed higher scores in processing speed that became similar to those of heterosexual men with aging (Faris, 2016). A meta-analysis revealed that gay men performed like heterosexual women in both male-favoring (e.g., spatial cognition) and female-favoring (e.g., verbal fluency) cognitive tests, while lesbians performed like heterosexual men only in male-favoring tests, with larger magnitudes for spatial abilities (Xu et al., 2017). Neuropsychological and psychological testing controversies highlighted problems when choosing the appropriate gender norms in transgender individuals as many tests had gender-based norms (Keo-Meier and Fitzgerald, 2017; Trittschuh et al., 2018). In addition, a call for a change in the culture of neurodisability and ableism emerged as recommendations were made to improve health care in LGBTQIA+ individuals (Moreno et al., 2017). Another line of research demonstrates that cross-sex hormone treatment affects cerebral tissue in transgender people using longitudinal MRI measurements of cortical thickness (Kilpatrick et al., 2019). Their study demonstrated that compared to controls, both transgender men and women showed significant decreases in the mesial prefrontal and parietal cortices.

There is still a small number of brain imaging studies *in vivo* in transgender people that are difficult to integrate because they differ in terms of techniques, research design, and samples (Kreukels and Guillamon, 2016). Also, there are different developmental trajectories confirmed by the fact that not all children with gender incongruence become transgender adolescents or adults. Conversely, not all transgender adults have been children with gender incongruence.

### Preparing for the future

The last period from 2021 to September 2023 included 119 records with different topics addressing neuroanatomical, hormonal, and functional differences. A distinguishing feature in the interpretation of findings is characterized by the inclusion of previously overlooked variables, such as minority stress. Studies also focused on body satisfaction and body ownership in transgender individuals of different ages. A large MRI study showed that transgender participants seemingly presented with their own unique brain phenotype and not only a male-female shift (Mueller et al., 2021). Neuroanatomical differences were also documented in a study with a neuroimaging-genetics dataset suggesting that genetic factors related to same-sex behavior may contribute to structural variation in certain brain structures (Abe et al., 2021). A study showed that, compared with cisgender individuals, transgender people showed lower cortical gyrification index limited to the occipito-parietal cortex and the sensory motor cortex regions, encoding own body image and body ownership (Wang et al., 2021). Another study in transgender youth showed that hormonal treatment was associated with significantly lower body image dissatisfaction and greater functional connectivity between the amygdala and ventromedial prefrontal cortex during a task designed to engage the amygdala (Grannis et al., 2023).

Additional research focused on the role of minority stress having an effect in emotion processing among transgender individuals in a study using fMRI and magnetic resonance spectroscopy (Kiyar et al., 2022). A meta-analysis of neuroimaging studies showed that minority stress associated with alterations within intrinsic connectivity networks was examined in only one study. Moreover, other studies were limited to investigating the neurobiological basis of sexual orientation (Nicholson et al., 2022). Some contributions focused on intersexuality including one study measuring the impact of HIV on brain health for racial/ethnic older adult LGBTQ people of color (Ramos, 2021). Further developments included a model of sexual differentiation where genes, hormones, and the environment act together in multiple parallel pathways leading to male or female phenotypes (Rouse and Hamilton, 2021).

From the point of view of neurocognitive disorders, a study showed that the prevalence of Alzheimer's disease and related dementias was higher in transgender adults as compared to cisgender adults (Guo et al., 2022). Recently, machine learning algorithms are being used to detect sexual orientation based on gray matter volumes with 62% accuracy and 92% with resting-state functional connectivity (Clemens et al., 2023), but this approach has been criticized in the prediction of gender identity with high risk of misleading conclusions (Wiersch et al., 2023). Researchers are now taking position in neuroimaging studies stating that their goal is not to uncover a mechanism that can be “fixed” to prevent gender diversity, but to make progress toward destigmatization, greater acceptance, and improved quality of life for individuals with diverse gender identities (Xerxa et al., 2023).

**Figure 2:**
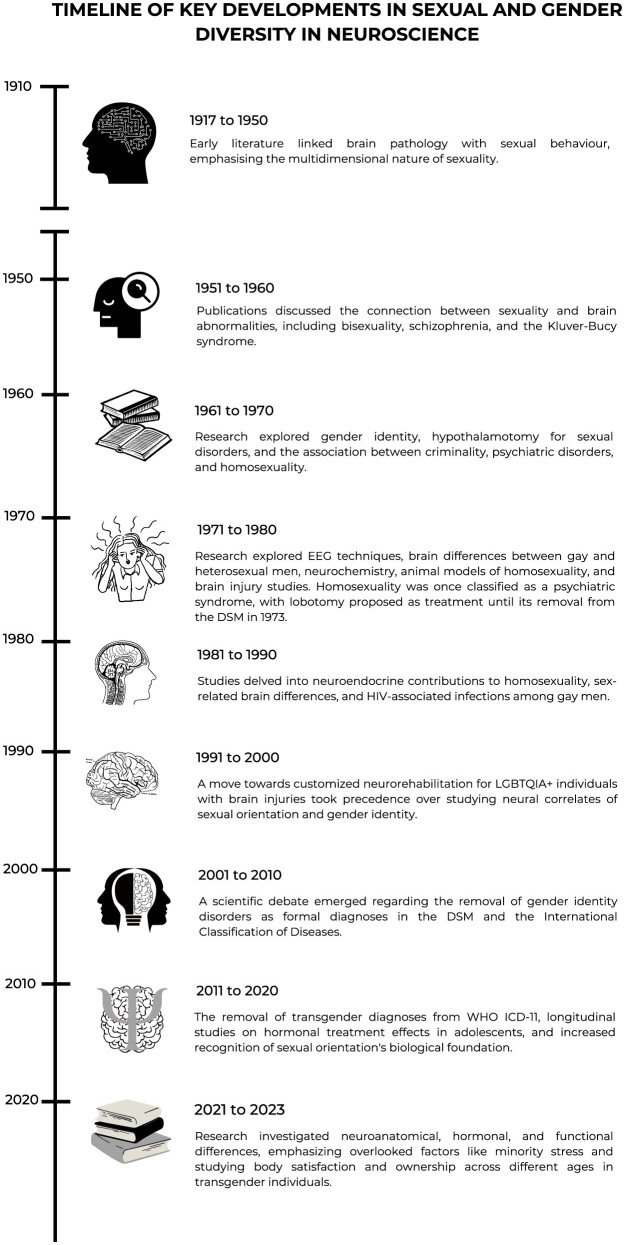
Timeline of key developments in sexual and gender diversity in neuroscience.

Having provided a historical overview and timeline, divided into decades, of key developments in sexual and gender diversity in neuroscience, also reflected in Figure 2, toward concluding the opinion paper, we now look to the future.

## Concluding remarks: the future of neuroscience of sexual and gender diversity

Any historic account is incomplete without asking whose histories are being told and by whom (Hegarty and Ruterford, 2019; Horne et al., 2019; Moreno et al., 2019). The selected research presented chronologically above may indeed suggest a White cisgender male, and a Northern American and British skew in who conducted the research and where it was published. Yet, this account demonstrates how competing theories and concepts were interpreted based on ideologies, values, and political opinions that have generally evolved throughout the 20^th^ and the first decades of the 21^st^ century. Unsurprisingly, some published research was subsequently retracted by editors reconsidering long discredited beliefs and unethical practice, such as conversion therapy; notably, some of these studies are kept in archives only for their historical value (i.e., see Glover, 1951).

Large parts of the scientific world have significantly shifted from an obsession with pathology and causation to one in which sexual and gender diversity is increasingly affirmed and even celebrated. However, it will serve us well to consider how some countries are on record for excluding candidates for psychiatric residences or doctoral programs based on their “militant homosexuality” (Moreno Robles, 1975; Kooden, 2021), and so-doing limited sexual and gender diversity perspectives informing scientific research in this field. Still today, some journals and scientific fields may unintentionally, or otherwise, operate from a hetero-cis-normative epistemological position (Pillay et al., 2022). Also in neuroscience, biased science, research neglect, and exclusion have had negative consequences leading to a narrow comprehension of the full spectrum of sexual and gender diversity. A clear example is the persistent neglect of lesbian, bisexual, and intersex concerns evidenced by the reduced number of studies targeting these specific communities, as compared to the large number of studies with gay and transgender individuals.

Throughout its brief history, neuroscience has been used to understand the biological foundations of sexual and gender expressions. While an invaluable contribution, unfortunately, this quest does not automatically lead to positive social changes. For example, a parallel can be established for skin color, the biological foundations of which have clearly been established (Naik and Farrukh, 2022). Yet, while race relations have changed dramatically in recent years, this has not prevented racism in our societies. Dated and unjustified beliefs, homo-, bi-, and transphobia, and even scientifically discredited practices, such as conversion therapies are not consigned to history and are difficult to eradicate and can perpetuate healthcare disparities (White and Chanoff, 2011; Academy of Science of South Africa, 2015).

And regardless of cumulative research demonstrating the multidimensional nature of human sexuality, very real health disparities exist among LGBTQIA+ individuals across their lifespans. The same applies to hetero-cis-normative assumptions as dangerous political and social determinants of health, and rigid conceptual binaries of, among others, heterosexual and homosexual, male and female, masculine and feminine, transgender and cisgender (Pillay et al., 2022). It is important to move away from a focus on etiology to a recognition of the effects of minority stress on the brain and on mental health (Edmiston and Juster, 2022). Avoiding a rigidly binary conceptualization of biological sex, either explicit or implicit (Rouse and Hamilton, 2021), and shifting away from research that focuses solely on the “etiology” or origins of LGBT identities (Edmiston and Juster, 2022) seem to be two promising approaches to push forward the neuroscience of sexual and gender diversity.

The historical overview presented in this opinion paper may assist scientists to reflect on how their research can maintain and/or reinforce stereotypes and harmful ideas regarding sexual and gender diversity. Explicit or implicit biases can contaminate research, amplify disparities, and translate into significant negative health consequences for LGBTQIA+ people. Given the social responsibility of science, researchers must clearly state the theoretical models behind the rationale of their studies and anticipate the consequences of their findings to reduce negative stereotypes, disparities, and stigma. As such, researcher reflexivity and justification ought to be encouraged by funding agencies supporting research on sexual and gender diversity. Ultimately, related research should serve to create an environment where LGBTQIA+ people feel comfortable disclosing their sexual orientation and gender identity to destigmatize care (Colin, 2015).

In including previously overlooked variables, such as minority stress, in the interpretation of neuroscientific findings, lessons are to be learnt from recent shifts to multisystemic, interpersonal, contextual, and affirmative understandings in LGBTQIA+ Psychology, also in relation to resilience science (Wilks et al., 2022). Notably, psychological science has been at the forefront of advancing affirmative practice guidelines development in many parts of the world to contribute to service provider cultural competence and ethical practice in working with LGBTQIA+ client populations (Horne et al., 2019; Moreno et al., 2019; Pillay et al., 2022; Wilks et al., 2022). Similarly, it will serve the neuroscientific field well to incorporate an intersectional lens more deliberately in future research, that is, discourses of capacity (and ableism), race (and racism), gender (and sexism and cisgenderism), class (and classism), and sexuality (and heterosexism) (Hegarty and Ruterford, 2019). There is also an urgent need to expand understandings of sexual and gender diversity from the non-WEIRD (Western, educated, industrialized, rich, and democratic) world (Henrich et al., 2010). Moving away from the binary model, the significant definitional shifts in understanding sexual orientation (Park, 2022), sexual orientation development and the science of sexual and gender fluidity (Diamond, 2020, 2021), ought to be considered. Also, to remain current, terminologies now employed in the healthcare of trans and gender-diverse people (Tomson et al., 2021) should be adopted.

Hegarty (2018, p. 99) posed the rhetorical question: “What discovery made us feel that neuroscience might be a politically progressive narrative in the decade of the brain?” As we look to the future, several questions preoccupy the authors. Given a historical focus on pathology, is there any value in applying neuroscientific research to “explain” the origins of sexual and gender diversity? Given the issue of replicability, are findings just isolated results? Beyond scientific knowledge, is this quest deprived of value and concrete positive repercussions for LGBTQIA+ people? From a translational perspective, what is the value of animal research, animal models and genetics in explaining sexual orientation or gender identity in humans? Is this still relevant to sexual and gender diversity and how can sociocultural factors be studied in animal models to mimic the human condition? In thinking about emerging technologies, how is artificial intelligence going to be used to increase predictions of sexual and gender identity in neuroscience? If we abandoned lobotomies to control sexual and gender diversity, could science be used to control the sexualities of individuals using more sophisticated ways, such as artificial intelligence, considering that such data will be feeding algorithms? How can researchers, scientists, and clinicians demonstrate responsibility and persist in the prevention of abuse and harm?

These are important questions that we do not have answers to currently. As we move forward respectfully and inclusively, we must ensure that the power of neuroscience is used for good and not harm. We, the authors, hold the conviction that studies on how stigma, stress, and strain shape the brain are of greater value to LGBTQIA+ communities than fixating and focusing on sexually dimorphic nuclei to explain why people are different. Rather, by showing how socially constructed pressures can impact neural functions, we provide the strongest possible evidence that the environment impacts the brain and that more progressive spaces can be promoted so that people can live authentic lives without prejudice. During this time of war, climate crisis, and pandemic recovery, we fear that conservative voices are becoming louder in their hate toward LGBTQIA+ people. With this in mind, it is important to build upon neuroscientific research on sexual and gender diversity in a manner that includes the unique lived experiences of the communities we study and serve.

Several efforts have been made in neuroscience resulting from a significant interaction between clinical observations, technical advancement, and social attitudes. Science is self-corrective, and neuroscientists are challenging erroneous views and beliefs limiting the credibility of their findings. Scientific transparency and the willingness to change attitudes toward sexual and gender minorities along with increased knowledge will enable more trustworthy results. Indeed, funding agencies are also playing their role introducing clear guidelines supporting equity, diversity, and inclusion in research.

## Footnotes

^1^While the authors acknowledge significant shifts in trans-related terminologies, those utilized at the time of the cited research have been retained.
